# Management of a Rare Mandibular Bilateral Glandular Odontogenic Cysts With Enucleation: A Case Report and Mini Review of Literature

**DOI:** 10.1155/crid/3879944

**Published:** 2026-03-06

**Authors:** Layla Hafed, Islam Ahmed Ghazy, Pasant Tarek Thakeb, Sally Ibrahim

**Affiliations:** ^1^ Oral Medicine and Diagnostic Science Department, Faculty of Dentistry, Saba University, Sana’a, Yemen; ^2^ Oral and Maxillofacial Surgery Department, Kafr Elshiekh General Hospital, Kafr Elshiekh, Egypt; ^3^ Consultant at Oromedlabs, Cairo, Egypt; ^4^ Oral and Maxillofacial Pathology Department, Faculty of Dentistry, Fayoum University, Fayoum, Egypt, fayoum.edu.eg

**Keywords:** bilateral, bone graft, enucleation, glandular odontogenic cyst, odontogenic cyst

## Abstract

Glandular odontogenic cysts (GOCs) are rare odontogenic cysts which exhibit distinctive histopathological characteristics and a notable tendency to recur. The radiographic features of GOCs are not specific to the condition. A definitive diagnosis of GOC can only be confirmed through histopathological examination, which identified a nonkeratinized squamous epithelial lining with nodular thickening, papillary projections, and mucous cells that contain mucous pools within the epithelium as well as or duct‐like structures (microcystic spaces). We report a rare case of bilateral GOCs located in the mandible treated successfully with simple enucleation and bone grafting with collagen membrane placement. A 31‐year‐old Kuwaiti male patient showed with a painless swelling on both sides of the mandible. Gave a history of swelling for 3–4 years subsided with antibiotics and anti‐inflammatories. An incisional biopsy was done and diagnosed as GOC. Surgical enucleation with bone curettage and bone grafting with a collagen membrane to reinforce the mandible was the best line of treatment based on the patient’s inquire. The enucleated tissue was submitted to histological examination, and confirming the previous diagnosis. The patient was followed up for 9 months with no recurrence; as well as a new bone formation was detected. Differentiation of GOC from other odontogenic cysts and tumors, or intraossous mucoepidermoid carcinoma, is crucial to avoid unnecessary aggressive treatment. More care should be given to report this rare entity with this presentation as bilateral GOCs.

## 1. Introduction

In the annum of 1987, Padayachee and Van Wyk [1] proposed the term “sialo‐” for multilocular cystic lesions that resembled botryoid odontogenic cysts but were distinguished by a glandular component, based on the presence of mucin in the cyst epithelium and the absence of a salivary gland. Shortly thereafter, in 1988, Gardner et al. [2] reported eight additional cases and formally named the entity the glandular odontogenic cyst (GOC).

GOC most commonly affects middle‐aged individuals [3] and demonstrates a slight male predominance [4]. Although it can occur in both jaws, the condition is observed more frequently in the mandible [5], particularly its anterior region [6]. Clinical presentations may include pain, swelling, or discharge; however, GOCs are often asymptomatic and discovered incidentally during routine radiographic examinations, especially in contexts of tooth eruption failure, missing teeth, or dental misalignment; however, large cysts can be associated with pain or paresthesia [7–9]. While benign, GOCs can be locally aggressive and have a notable recurrence rate, particularly the multilocular variant [4, 7, 10]. The lesion radiographically presents as a well‐defined unilocular or multilocular radiolucency [8, 9]. Associated findings can include cortical bone destruction, root resorption [4], and occasionally an impacted tooth within the cyst cavity [11, 12]. Computed tomography (CT) is recommended for diagnosis, surgical planning, and follow‐up [13].

Histologically, GOC is characterized by a thin, nonkeratinized squamous epithelial lining. This lining features papillary projections, nodular thickenings, mucous (goblet) cells containing epithelial mucin pools, and glandular or duct‐like microcystic structures. A superficial layer of cuboidal or columnar cells is also typically present [4]. The most frequent treatment is enucleation with peripheral curettage or marginal excision. However, some experts advocate for marginal resection as a more reliable option due to the potential for recurrence after enucleation and curettage [3].

This study aims to present an exceptional case of a bilateral GOC located in the mandible. Furthermore, this report constitutes the third documented instance worldwide of a bilateral GOC treated successfully with enucleation and bone grafting with collagen membrane placement. being successfully treated with enucleation.

## 2. Case Report

In the present report, a 31‐year‐old, Kuwaiti male patient, was referred by endodontics to a private clinic (Oral Surgery Clinic, Cairo, Egypt) in October 2024 with painless swelling on both sides of his mandible. An endodontist found the lesion during routine radiography while the patient was receiving dental treatment. When asked about the history of the swelling, the patient revealed that he had experienced painless swelling for 3 to 4 years, which had been relieved by antibiotics and anti‐inflammatory medications at that time. The patient is a smoker; however, he is medically free with no familial history of similar lesions.

**Figure 1:**
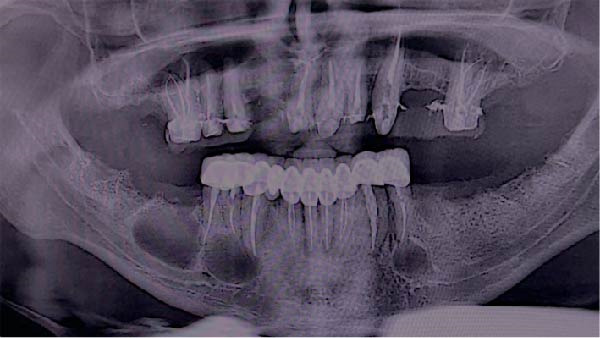
Preoperative panoramic radiograph showing bilateral well‐defined radiolucent lesions with radiopaque margins.

A panoramic radiograph demonstrated a well‐defined, unilocular radiolucency present on both sides of the mandible, where the lesion on the right side appeared as two separate lesions. The lesion on the left side is related to endodontically treated second premolar. However, lesions on the right are related to the endodontically treated canine, first and second premolars as well as the area of the missing first molar. There was no evidence of roots resorption or teeth displacement. All lesions were surrounded by a thin and intact cortical bone layer (Figure 1). The provisional diagnosis was radicular inflammatory cysts, odontogenic keratocytes and GOCs. Patient informed consent was signed for biopsy and surgical intervention as well as publication of the case report with the accompanying images. Incisional biopsies were done from the three lesions and submitted to histopathological examination. Microscopic examination of hematoxylin and eosin (H&E) stained sections of whole lesions revealed a cystic cavity lined by variable thickness of nonkeratinized stratified squamous epithelium with surface columnar cells with areas of hobnail appearance. The lining showed areas of mucous‐secreting cells forming duct‐like structures and cystic spaces. Areas of nodular thickness and papillary projects were also noted. The underlying connective tissue showed numerous cholesterol clefts and varying degrees of inflammation. All the right and left specimens shared the same criteria which are consistent with GOC.

**Figure 2:**
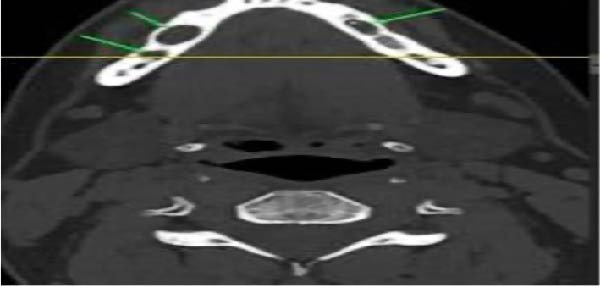
Preoperative computerized tomography (CT) scan (axial cut) revealed bilateral radiolucenct lesions.

CT scan of the facial bone (Figure 2) revealed two right intramedullary mildly expansile cystic lesions measuring 10 mm x 5 mm and 8 mm x 4 mm and one left small intramedullary lesion measuring 5 mm x 3 mm with intact bony boundaries.

**Figure 3:**
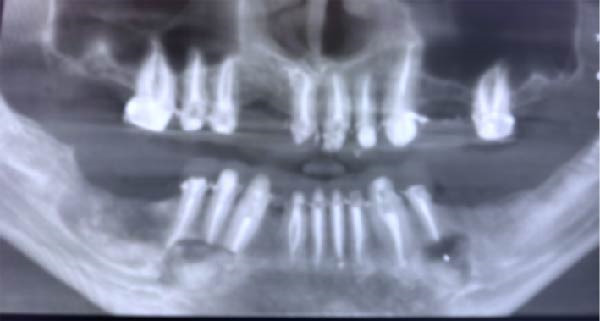
Panoramic radiograph after lesions enucleation, placement of the collagen membrane and retreatment of the whole lower teeth.

**Figure 4:**
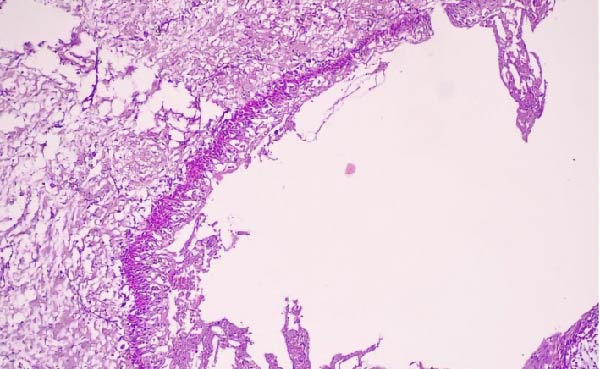
Photomicrograph of right GOC showing a cystic cavity lined by nonkeratinized stratified squamous epithelium showing papillary projections (H&E; original magnification ×40).

**Figure 5:**
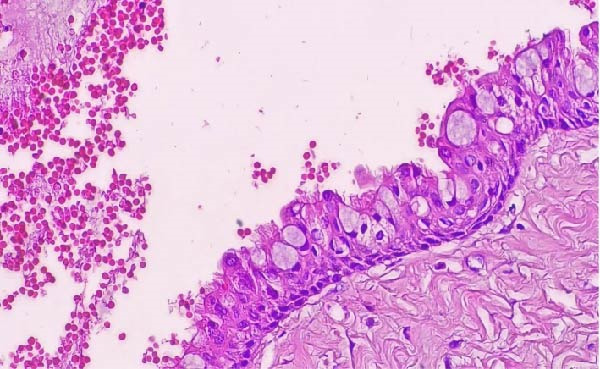
Higher magnification of the previous photomicrograph showing the stratified squamous epithelium which contains goblet cells, glandular structures, and intraepithelial cysts (H&E; original magnification ×400).

**Figure 6:**
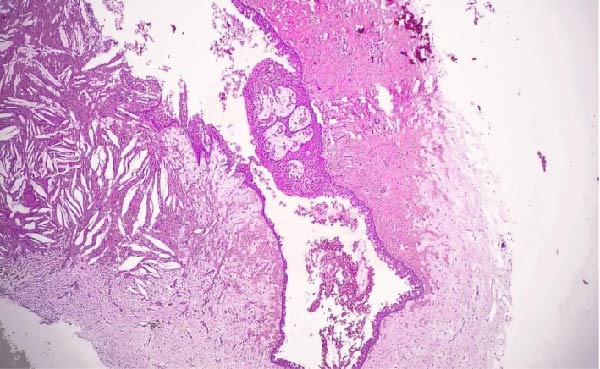
Photomicrograph of the left GOC showing a cystic cavity lined by variable thickness stratified squamous epithelium that shows focal nodular thickening with microcysts and duct‐like structure. The connective tissue showed varying degrees of inflammation, with numerous cholesterol clefts (H&E; original magnification ×100).

**Figure 7:**
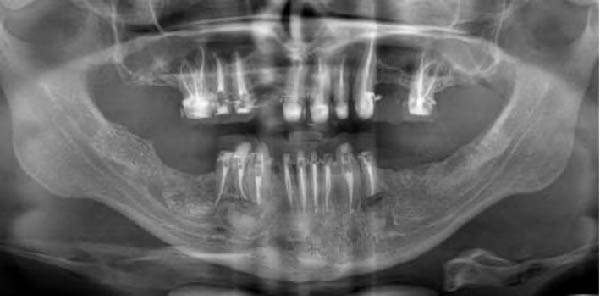
Follow‐up panoramic radiograph after 9 months showing bone formation at the sites of the lesions.

Consequently, proposed surgical enucleation of the three lesions with marginal removal of the lesion and then bone graft (Zeno graft bone grafting 4 gm on each side, OneXeno Graft, Germany) mixed with autogenous bone grafting collected from the retromolar area bone was used to fill the cavity left by the lesion and reinforce the mandible alongside a collagen membrane to cover the bone (Bioguard 15 mm x 25 mm, Russia) for each side (Figure 3). That was after going over various treatment options with the patient, including therapy involving surgical removal and replacement with plates and screws; however, the patient declined this option. The excised specimens were also sent for histopathological investigation to confirm the diagnosis of GOC (Figures 4–6). Follow‐up after 9 months postoperatively showed that the healing process was uneventful, and there was no evidence of recurrence. New bone formation was also noted in the follow‐up radiographs (Figure 7).

## 3. Discussion

The GOC is a developmental odontogenic cyst, accounting for less than 0.5% of all jaw cysts, with no clear gender predilection, and an average patient age of 50 years [14]. GOC lacks a pathognomonic radiographic appearance [5], which often leads clinicians to initially misdiagnose a radiolucent lesion as a more common condition, such as a dentigerous or radicular cyst [11]. These cysts exhibit a strong preference for the mandible, occurring there at a ratio of ~4:1 compared to the maxilla, with a particular predilection for the anterior regions [5]. Bilateral presentation of GOC is exceptionally rare, with only two previous cases documented in the literature [15, 16]. The present case is also bilateral, with lesions located in the mandibular premolar area on one side and the premolar–molar area on the other.

As shown in Table 1, the clinicopathological and radiographic data from the two documented cases reveal that all reported patients were middle‐aged males, ranging from 29 to 40 years [15, 16], which aligns with the current case. Furthermore, all previously reported lesions were expansile and asymptomatic [15, 16], consistent with this case where the patient presented with bilateral painless swellings.

**Table 1:** Clinicopathological and radiographic data of the two documented cases.

Study	Age	Gender	Site	Clinical picture	Radiographic picture	Treatment	Follow‐up
Akkaş et al. [16]	40 years	M	Bilateral posterior mandible	The right mandibular canine had preserved vitality. There was a preserved sensation of the lower lip bilaterally.	WD, UL, and RL present on both sides of the body of the mandible	Peripheral osteotomy with Carnoy’s solution, followed	Up 36 months. Uneventful with no recurrence.

Amberkar et al. [15]	29 years	M	Bilateral posterior maxilla	Pain that worsened when chewing hard food.	Periapical RL	Curettage, enucleation, margin dissection, and local block excision.	Unclear

Abbreviations: Cm, centimeter; F, female; M, male; NM, not mentioned; RL, radiolucent; UL, Unilocular; WD, well‐defined; yrs, years old.

Radiographically, GOC typically presents as a well‐defined unilocular or multilocular radiolucency. Similar to our case, the previously documented bilateral GOCs were also reported as well‐defined, unilocular lesions [15, 16].

All documented cases, including the present one, share the histopathological criteria established by the WHO 2022 classification [14]. The histology resembles salivary gland or glandular differentiation, characterized by a cystic cavity lined by epithelium of variable thickness featuring whorled epithelial thickenings. Key features include hobnail cells (cuboidal cells on the luminal side) with possible findings of clear cells, intraepithelial microcysts, papillary projections, cilia, and mucous cells. Consequently, careful microscopic examination is essential to confirm a GOC diagnosis, as its histopathological criteria and aggressive behavior, are similar to low‐grade intraosseous mucoepidermoid carcinoma (IMEC) [17]. In a recent documented case serious, studied the histologic and demographic findings of both IMEC and IMEC with GOC features. It seems highly possible that a GOC may eventually develop into IMEC. Therefore, long‐term follow‐up is essential to prevent this progression [18]. According to the recent WHO Classification [14], the diagnosis of GOC rests on its distinct histopathological characteristics and can be established reliably without immunohistochemistry (IHC). Although IHC may assist in the differential diagnosis, particularly between GOC and low‐grade mucoepidermoid carcinoma, it was not employed in this case report. The definitive histopathological features include nonkeratinized stratified squamous epithelium of variable thickness, intraepithelial microcysts or duct‐like spaces, hobnail cells, and epithelial plaques, this was also emphasized in a recently published case series [19].

The treatment for GOC involves either enucleation with curettage or marginal resection [20]. While some authors suggest conservative treatment leads to a higher recurrence rate of ~21.6%, a review of 169 cases by Chrcannovic et al. [21] found no significant difference in recurrence between enucleation and resection, ultimately favoring enucleation/curettage for its superior recovery and rehabilitation outcomes. One of the previously documented bilateral GOCs was successfully treated with enucleation and showed no recurrence after 36 months of follow‐up [16], similar to the present case. For our patient, a decision was made for clinical and radiographic follow‐up, which at 9 months postoperation showed complete healing. The follow‐up period for the second documented bilateral case, however, was not specified [15].

In conclusion, this report describes the third documented case of a bilateral GOC. The clinical and radiographic features of GOC are often nonspecific, mimicking more common lesions such as radicular cysts, odontogenic keratocysts, or other odontogenic tumors. As demonstrated, a definitive diagnosis relies entirely on meticulous histopathological examination, which can be challenging due to its resemblance to other entities, particularly low‐grade central mucoepidermoid carcinoma. This distinction is critical as it drastically alters the surgical plan, long‐term management strategy, and the monitoring for potential recurrence.

## Author Contributions

**Layla Hafed and Sally Ibrahim:** data analysis and editing the manuscript. **Islam Ahmed Ghazy and Pasant Tarek Thakeb:** data collection and drafting of the manuscript.

## Funding

No funding was received.

## Consent

The authors declare that informed consent was signed for biopsy and surgical intervention as well as publication of the case report with the accompanying images. A copy of the written consent is available upon request by the corresponding author.

## Conflicts of Interest

The authors declare no conflicts of interest.

## Data Availability

The data that support the finding of the case report are available within the manuscript.
